# Varicose veins in hairdressers and associated risk factors: a cross-sectional study

**DOI:** 10.1186/1471-2458-14-885

**Published:** 2014-08-28

**Authors:** Chao-Lin Chen, How-Ran Guo

**Affiliations:** Chest Hospital, Ministry of Health and Welfare, Tainan, Taiwan; Department of Occupational and Environmental Medicine, National Cheng Kung University Hospital, Tainan, Taiwan; Department of Environmental and Occupational Health, National Cheng Kung University, 138 Sheng-Li Road, 70428 Tainan, Taiwan

**Keywords:** Varicose veins, Hairdressers, Occupation, Standing, Epidemiology

## Abstract

**Background:**

Varicose veins (VV) cause not only cosmetic problems but also clinical symptoms such as pain of the affected limbs. Whereas an occupation associated with orthostasis has been recognized as a risk factor of VV, epidemiological studies on working populations are limited. We conducted a study to identify the risk factors of lower limb VV among hairdressers in Taiwan and evaluate their effects, with a focus on long-term standing at work.

**Methods:**

We recruited participants from members of a hairdressers union in southern Taiwan and conducted a questionnaire survey. Data on demographic characteristics, body weight and height, work history, medical history, and other potential related factors were collected from each participant.

**Results:**

A total of 182 hairdressers participated in the survey, and 44 (24.2%) had lower limb VV. Uni-variate analyses showed that hairdressers with lower limb VV tended to be older (49.3 *vs.* 44.7 years, *p* = 0.032), have a family history of VV (25.6% *vs.* 9.9%, *p* = 0.011), doing housework in standing position (86.4% *vs.* 71.0% , *p* = 0.042), have a longer work history (30.5 *vs.* 24.0 years, *p* = 0.005), and stand longer at work (213.9 *vs.* 176.0 hour/month, *p* = 0.008). In multivariate analysis, in hairdressers ≤ 45 years old, the only significant risk factor was a family history of VV (odds ratio [OR] = 11.9, 95% confidence interval [95% CI] = 1.1-133.5). In hairdressers > 45 years old, the risk factors included standing working for > 260 hours per month (OR = 31.8, 95% CI = 1.8-566.5) and working as a hairdresser for > 30 years (for 31–42 years, OR = 10.9, 95% CI = 1.6-73.8; for ≥ 43 years, OR = 12.0, 95% CI = 1.6-88.5).

**Conclusions:**

In hairdressers ≤ 45 years old, family history of VV is a major risk factor for developing lower limb VV, while in those who are > 45 years old, the effects of occupational risk factors are more prominent.

## Background

Varicose veins (VV) may cause discomfort of the affected limbs and cosmetic problems, which often need surgery or other invasive treatments, consuming health care budget [1] and reducing productivity of the workers. This illness was due to venous valves insufficiency, with age as an aggravating and risk factor and family history as an important risk factor [2]. Other risk factors include female gender, the number of children given birth, body weight, constipation, and history of venous thrombosis [2–7]. Prolonged standing at work had been suspected as an aggravating or risk factor of VV of lower limbs, but findings in previous studies were not consistent [4].

For hairdressers, prolonged standing is an inevitable part of work. A survey on hairdressers in Tehran, Iran showed a VV prevalence rate of 35.62% [8], but the relationship between prolonged standing at work and VV was not studied. Therefore, we conducted a study in southern Taiwan to evaluate whether prolonged standing at work can increase the risk of developing VV.

## Methods

### Data collection

We recruited participants from members of a local union of hairdressers in southern Taiwan and conducted a questionnaire survey. The questionnaire had been validated by an expert panel of four researchers before its use. The panel included an occupational medicine specialist, an industrial hygienist, an occupational nurse, and a surgeon, and each member reviewed the draft independently. The first author (C-L Chen) finalized the questionnaire according to the comments from the panel. Data on demographic characteristics, body weight and height, work history, daily work hours (including hours of standing), monthly work days, current morbidity, medical history (including VV, deep venous thrombosis, constipation [defined as weekly defecation less than 3 times], surgery, and major trauma), family history (including family history of VV), and activities after work (including regular sport activities, part-time job, and doing housework in standing position) were collected from each participant by the use of self-administered questionnaire.

To evaluate the effects of prolonged standing at work, we calculated the hours of standing in working each month as daily standing work hours multiplied by monthly work days. For example, an employed hairdresser who worked in standing position about 7 hours a day for 24 day a month (taking every Sunday and every other Saturday off as the maximum work days allowed by law in Taiwan) had 7 × (30–4 [Sundays] - 2 [Saturdays]) = 168 hours of standing in working each month. Likewise, a self-employed hairdresser who worked in standing position about 4 hours a day for almost every day (30 days a month) had 4 × 30 = 120 hours of standing in working each month.

### Data analysis

We evaluated differences in continuous variables between those with and without lower limb VV using the Student’s *t* test or Mann–Whitney U test and differences in categorical variables using the Chi-square test or Fisher’s exact test. We conducted further analyses using multivariate logistic regressions. The models included variables that appeared to be significant predictors of lower limb VV in the uni-variate analyses. According to the results of initial analyses, we divided the participants into two age groups (≤45 years old and > 45 years old) and conducted separate analyses to adjust for the effects of age and demonstrate its effect modification of other risk factors. All statistical analyses were performed using SPSS 12.0 (SPSS Inc., Chicago, IL, USA) at the two-tailed significant level of 0.05.

### Ethics statement

This study was ethically approved by the Grant Review Committee of the Council of Labor Affairs of the Taiwan government. At the time when this study was conducted, the Regulations on Human Trials have not been implemented by the Taiwanese government. We conducted the study according to the principles expressed in the Declaration of Helsinki and analyzed the data anonymously. All participants submitted a written informed consent to participate in the study.

## Results

A total of 182 hairdressers participated in the study, including 166 women (91.2%), 15 men (8.2%), and 1 who did not provide information on the gender. The average age was 45.8, but 11 participants did not provide information on the age. There were 44 cases of lower limb VV, corresponding to a prevalence of 24.2%, and the average duration of the illness was 16.2 years (standard error [SE] = 1.7 years).

In comparison with hairdressers without the disorder, those who had lower limb VV had a longer work history (the number of years of working as a hairdresser was 30.5 *vs.* 24.0 years, *p* = 0.005), an older age (49.3 *vs.* 44.7 years, *p* = 0.032), and more hours of standing in working as a hairdresser each month (213.9 *vs.* 176.0 hours, *p* = 0.008) (Table 1). The body mass index (BMI), number of childbirths, and number of hours doing housework in standing position did not appear to be significant predictors of lower limb VV (all with *p* > 0.05). Among the categorical variables, significant predictors included having a family history (parents, grandparents and siblings) of VV (25.6% *vs.* 9.9%, *p* = 0.011) and doing housework in standing position (86.4% *vs.* 71.0%, *p* = 0.042) (Table 2). Gender, age stratum, having constipation, regular exercise, and habits of smoking and drinking did not appear to be significant predictors of lower limb VV (all with *p* > 0.05). We also tried analyzing the number of childbirths as a categorical variable (0, 1, 2, 3, and > 3), but it still did not appear to be a significant predictor.

**Table 1:** **Comparison of continuous variables between hairdressers with and without lower limb varicose veins (VV)**

Variable	With VV	Without VV	***p***value*
	Mean (SE)	Mean (SE)	
Work history (year) (n =167)	30.5 (1.9)	24.0 (1.2)	0.005
Age (year) (n =171)	49.3 (1.6)	44.7 (1.1)	0.032
Body mass index (kg/m^2^) (n = 170)	23.5 (0.6)	23.0 (0.3)	0.407
Monthly standing work hours (hour) (n =135)	213.9 (11.3)	176.0 (7.6)	0.008
Childbirth (time) (n = 126)	2.46 (0.2)	2.14 (0.1)	0.105
Standing housework** (hour/week) (n =109)	12.3 (2.1)	10.1 (1.2)	0.334

*two-sample t test; **for those who do any standing housework.

**Table 2:** **Comparison of categorical variables between hairdressers with and without lower limb varicose veins (VV)**

Variables	With VV	Without VV	***p***value
	N (%)	N (%)	
Total	44 (24.2)	138 (75.8)	
Sex			0.529*
Women	42 (25.3)	124 (74.7)	
Men	2 (13.3)	13 (86.7)	
Age (year)			
≤ 35	3 (9.4)	29 (90.6)	
36-45	12 (48.8)	31 (51.2)	
46-55	10 (23.2)	33 (76.8)	
> 55	15 (34.9)	28 (65.1)	
Family history of VV			0.011**
Yes	11 (47.8)	12 (52.2)	
No	32 (22.7)	109 (77.3)	
Constipation			0.192**
Yes	6 (37.5)	10 (62.5)	
No	38 (22.9)	128 (77.1)	
Regular sport activities			0.607**
Yes	21 (26.6)	58 (73.4)	
No	23 (23.2)	76 (76.8)	
Smoking			0.764*
Yes	3 (18.8)	13 (81.2)	
No	41 (25.3)	121 (75.7)	
Drinking			0.564*
Yes	3 (17.6)	14 (82.4)	
No	41 (27.0)	111 (73.0)	
Standing housework			0.042**
Yes	38 (29.0)	93 (71.0)	
No	6 (13.6)	38 (86.4)	

*Fisher’s exact test; **Pearson’s chi-square test.

When we divided participants into four age groups (≤35 years, 36–45 years, 46–55 years, and > 55 years), we found that in the age group 46–55 years, hairdressers with lower limb VV had a longer work history (33.6 *vs.* 29.0 years, *p* = 0.044) (Table 3). Differences in work history in the other age groups did not reach statistical significance (all with *p* > 0.05), indicating effect modification, and therefore we conducted further stratified analyses by age and use 45 years old as the cut-off.

**Table 3:** **Lower limb varicose veins (VV) and work history by age group**

Age (year)	Work history				***p***value*
	With VV		Without VV		
	N	Year (SE)	N	Year (SE)	
≤ 35	3	10.7 (2.7)	29	8.7 (1.2)	0.538
36-45	12	20.8 (2.4)	31	22.4 (1.2)	0.989
46-55	10	33.6 (1.7)	33	29.0 (1.4)	0.044
> 55	15	41.1 (1.9)	28	35.7 (2.6)	0.591

*Mann–Whitney U test.

In the multivariate analyses, a family history was the only significant predictor of lower limb VV identified in participants ≤ 45 years old in our study (OR = 11.9, 95%CI = 1.1-133.5) (Table 4). In the older participants (>45 years old), significant predictors included standing at work 261–360 hour/month (OR = 31.8, 95% CI = 1.8-566.5) and working as a hairdresser for > 30 years (31–42 years, OR = 10.9, 95% CI = 1.6-73.8; 43–57 years, OR = 12.0, 95% CI = 1.6-88.5), but a family history was not a significant predictor after adjusting for other variables (OR = 1.6, 95% CI = 0.4-7.1) (Table 5).

**Table 4:** **Multivariate logistic regression for hairdressers of age ≤ 45 year-old**

	N	Odds ratio	95% confidence interval
Monthly standing work hours			
6-120	11	1.0	
121-168	7	2.4	(0.1-55.6)
169-208	16	2.0	(0.2-24.2)
209-260	13	0.5	(0.0-11.3)
261-360	10	5.2	(0.4-75.8)
Number of years as a hairdresser			
0-9	20	1.0	
10-22	19	0.6	(0.1-5.3)
23-33	18	2.7	(0.3-27.4)
Doing housework in standing position			
No	24	1.0	
Yes	33	1.5	(0.2-10.2)
Family history of varicose veins			
No	52		
Yes	5	11.9	(1.1-133.5)*

*p < 0.05.

**Table 5:** **Multivariate logistic regression for hairdressers of age > 45 year-old**

	N	Odds ratio	95% confidence interval
Monthly standing work hours			
25-120	11	1.0	
121-168	13	9.7	(0.7-132.5)
169-208	12	7.4	(0.6-89.9)
209-260	10	6.4	(0.5-86.2)
261-360	9	31.8	(1.8-566.5)*
Number of years working as a hairdresser			
0-30	20	1.0	
31-42	18	10.9	(1.6-73.8)*
43-57	17	12.0	(1.6-88.5)*
Doing housework in standing position			
No	8	1.0	
Yes	47	0.9	(0.1-7.6)
Family history of varicose veins			
No	42	1.0	
Yes	13	1.6	(0.4-7.1)

*p < 0.05.

## Discussion

### Prevalence

The reported prevalence rates of VV differed enormously in previous studies, ranging from 2% to 56% in men and 1% to 73% in women [2, 3], probably affected by age, gender, race, and case definition. Of the studies conducted in Asia, one on 541 Japanese women observed a prevalence rate of 45%, including the mildest to the worst type of VV [5]. A study in the Shanghai area of China on 30712 workers more than 15 years old in various industries observed a prevalence rate of 8.39% (N = 2577) of lower limb VV [6]. The 24.2% prevalence rate of lower limb VV in hairdressers in our study was within the reported range.

### Family history of varicose veins

Our study found that having a family history of VV was a risk factor in the younger group (≤45 years old), but the effect was not statistically significant in the older group (>45 years old). Similar results was observed in a study of 541 Japanese women, which found a greater proportion of having a family history in the younger patient group (25–39 years old, 59%) than in the older patient group (>60 years old, 27%) [5]. Some other studies also showed that a family history of VV was a major risk factor [7, 9–11], and a study conducted by Cornu-Thenard et al. demonstrated that the risk increased dramatically in relation to the number of parents with VV [12]. Accordingly, we inferred that having a family history is a major risk factor of lower limb VV in the younger population. Because it is feasible to stop people with a family history of VV from working as a hairdresser or to implement protective measure since the early stage of their careers, findings in our study may help lowering the prevalence of lower limb VV in hairdressers.

### Gender, body mass index, constipation, pregnancy, exercise, smoking, and drinking

Previous studies have identified female gender [7, 9, 10, 13, 14] and obesity [7, 9, 13] as risk factors of VV, and other reported factors included previous pregnancies [7, 10, 15–17], race [14], oral contraceptives [11, 16], trauma or injuries to lower limbs [9], constipation [7, 16], and lower levels of activity [13] such as exercising less than once per week [15]. However, the effects of BMI, gender, times of childbirth, constipation, and regular exercise did not reach statistical significance in our study (Tables 1 and 2). The published data on the effects of smoking and drinking are limited. A large study on 1806 patients of lower limb venous insufficiency (not just VV) found that smoking more than 10 cigarettes per day was associated with a higher prevalence of venous insufficiency, but smoking less was not; none of the other factors related to smoking (tobacco quality, inhalation of smoke, use of filter, duration of smoking, etc.) was an independent predictor [18]. Alcohol abuse was not an independent risk factor for venous insufficiency in that study [18]. A study on VV exclusively, however, did not find smoking as a risk factor, but found a significant protective effect (OR = 0.50) associated with moderate alcohol consumption (≤1 drink/day) [19]. As the prevalence of both smoking and drinking is low in Taiwanese women and most hairdressers in Taiwan are women, the prevalence of both smoking and drinking was low in our participants. Neither factor was found to have a significant association with VV in our study.

### Work history

Aging has been recognized as a major risk factor of VV [2, 3, 7, 9, 10, 14, 20], but it usually correlates well with the length of work history. In our study, we tried to solve this problem by using stratified analyses according to age. We found the lengths of work history were similar (2 years apart or less) between participants with and without lower limb VV in the two younger age groups (≤35 years and 36–45 years), but the difference was statistically significant in the 46–55 year-old group (a difference of 4.6 years). In the oldest group (≥55 years), while a larger gap (5.4 years) was observed, the difference did not reach statistical significance, most likely due to the larger SE because of the larger age range covered in this group (Table 3). Therefore, in the multivariate analyses, we put the two older groups together and combined the two younger groups. As a result, we found that a long work history (>30 years) was a significant risk factor in the older age group (>45 years) (Table 5), but not in the younger age group (≤45 years) (Table 4).

### Prolonged standing at work and doing housework

The effect of prolonged standing at work is the primary focus of this study. We found monthly standing work hours (*p* = 0.008) and doing housework in standing position (*p* = 0.042) were related to lower limb VV, but not weekly housework hours (*p* = 0.334).

A study conducted by Krijnen et al. [21] on 387 male European workers showed that the number of years of working in standing position was associated with the severity of chronic venous insufficiency, indicating working in standing position is an aggravating factor of lower limb VV. A study of female cotton workers also found a greater prevalence of VV in those who worked standing up [22]. In our study, after adjustment for other risk factors, the effect of prolonged standing at work did not reach statistical significance until standing for more than 260 hours each month in the older group. In the younger group, while standing for more than 260 hours each month was also associated with a higher risk (OR = 5.2), the effect did not reach statistical significance, most likely due to insufficient statistical power (Table 4). The dose–response relationship between the number of hours standing at work and prevalence of lower limb VV was not obvious, and this might be attributable, at least in part, to a healthy worker effect [23], because some of the workers might choose to stand less often or even leave the job after acquiring VV.

After interviewing 2939 men and 2708 women aged 20–59 years old, a prospective study in Denmark found that during the 12 years of follow up, jobs requiring prolonged standing or walking were associated with a relative risk (RR) of 1.75 in men and 1.82 in women for hospitalizations due to VV [24, 25]. Similar results were observed in a cross-sectional study in Germany which examined 9935 civil servants and found that after adjusting for age and gender, the OR for VV was 2.2 for standing at work [11]. A study in Austria on 209 hospital staff found that staffs with chronic venous disease stood longer at work, 4.4 *vs.* 2.7 hours per day in men and 3.9 *vs.* 3.2 hours per day in women [26]. The Framingham Study in the US observed a higher incidence of VV in women who reported spending 8 hours or more on sedentary activities (sitting or standing) than those who spent 4 hours or less a day (*p* < 0.05) [13]. In addition, a study of female workers in a large department store found a higher prevalence rate of severe VV among those who standing at work [17]. In summary, there are sufficient evidences supporting that prolonged standing at work or home is associated with the occurrence of lower limb VV.

### Methodological consideration

Our study was based on self-administered questionnaires, and therefore over- or under-reporting of VV was possible. However, since the investigators had no influence on the employment of participants or their employers, there were no remarkable reasons for over- or under-reporting of VV. Another potential limitation of using a questionnaire to assess VV is the validity of the self-reported condition. Laurikka et al. [27] studied this issue by comparing the results of mailed questionnaires to those of clinical examination with Doppler sonography and found a sensitivity of 0.92 and a specificity of 0.93; the estimated sensitivity and specificity for blue-collar workers were up to 0.96 and 0.94 respectively. In addition, the relatively small number of participants made further stratification of the study population difficult and thus limited our evaluations of more potential risk factors at the same time.

As the working days per month and the number of hours standing at work varied a lot, the hours of standing in working each month had a wide large. Hairdressers have different ways to do the same task. For example, some cut hairs in standing position most of the time while others do it in sitting position most of the time. In addition, the job contents varied: entry-level hairdressers usually spend a lot of time in washing hairs while senior hairdresser often spend most of the time cutting hairs. Furthermore, some hairdressers have good business and work more than 10 hours a day with little time to rest, while some others sit in the shops for long hours just waiting for customers. Therefore, there were many combinations of the number of work days per month and the number of hours standing at work per day, and we did not have enough participants in each situation for further analyses. In other words, we were unable to evaluate whether the number of work days per month, the number of hours standing at work per day, or both really mattered. Although the two critical variables—family history and hours of standing in working—had relatively large numbers of missing data information which reduced the statistical further, the effects still reached statistical significance.

## Conclusion

In hairdressers ≤ 45 years old, family history of VV is a major risk factor for developing lower limb VV, while in those who are > 45 years old, the effects of occupational risk factors are more prominent. Therefore, preventive measures such as compression stockings and minimizing prolonged standing are recommended for workers with personal risk factors.

## Abbreviations

BMI: Body mass index
CI: Confidence interval
OR: Odds ratio
RR: Relative risk
SE: Standard error
VV: Varicose veins.
